# Impact of Universal Hepatitis B Vaccination on Prevalence, Infection-Associated Morbidity and Mortality, and Circulation of Immune Escape Variants in Russia

**DOI:** 10.1371/journal.pone.0157161

**Published:** 2016-06-09

**Authors:** Vitalina V. Klushkina, Karen K. Kyuregyan, Tatiana V. Kozhanova, Oksana E. Popova, Polina G. Dubrovina, Olga V. Isaeva, Ilya V. Gordeychuk, Mikhail I. Mikhailov

**Affiliations:** 1 Department of Viral Hepatitis, Chumakov Institute of Poliomyelitis and Viral Encephalitides, Moscow, Russian Federation; 2 Department of Epidemiology and Evidence-Based Medicine of Prophylactic Medicine Faculty, I.M. Sechenov First Moscow State Medical University, The Ministry of Heath of the Russian Federation, Moscow, Russian Federation; Saint Louis University, UNITED STATES

## Abstract

Vaccination of newborns against hepatitis B (HB) was introduced in Russia in 1998. Since then the incidence of acute HB has rapidly declined. However, prevalence of chronic HB remains stable. The aim of this study was to estimate the effect of vaccination on HBV-associated morbidity, and to assess the prevalence of HBV immune escape variants after 10 years of vaccination.

**Methods:**

6,217 sera samples collected from volunteers in six epidemiologically different regions of Russia were tested for serological and molecular markers of HBV infection. A mathematical model developed by the U.S. Centers for Disease Control and Prevention was used to estimate the effect of vaccination and birth dose coverage on the incidence of HB and adverse outcomes of infection.

**Results:**

Prevalence of HBsAg in the study population varied from 1.2% to 8.2%; anti-HBc detection rates were 13.0–46.2%. HBsAg detection rates in epidemiologically significant cohorts were 0.6–10.5% in women of childbearing age; 0–2.4% in children ≤5 years old; 1.9–8.1% in adults ≥30 years old. Mathematical modeling demonstrated that the current 96.1–99.6% level of birth dose coverage increased the effectiveness of vaccination 10–21 times compared to 50% and 0% birth dose coverage scenarios. HBV DNA was detected in 63 sera samples. The frequency of amino acid substitutions in HBsAg was 38% (24/63). Only in 3% (2/63) the mutations were within the *a*-determinant of HBsAg (M133T and G145S, one case each). None of the identified mutations eluded HBsAg detection, since all these samples tested positive for HBsAg by commercial ELISA.

**Conclusion:**

Despite a significant decline in acute HB incidence after the introduction of universal vaccination, many undiagnosed potential sources of infection remain. Low prevalence of HBV immune escape variants is a favorable predictor of vaccine effectiveness in the future.

## Introduction

Today, viral hepatitis B (HB) is a vaccine-preventable disease. Safe and effective vaccines became available for mass use in 1981, but during the next 10 years, in many countries HB immunization was carried out only in cohorts at high risk of contracting HB. This strategy had no significant impact on general prevalence or hepatitis B virus (HBV) infection. In 1992, the World Health Organization recommended vaccination of all newborns and children under 1 year old in combination with vaccination of adolescents [1].

Russia joined these efforts, having experienced a significant increase in infection rates per 100,000 people since 1993 (1993: 22.4, 1994: 26.8, 1995: 35.4, 1996: 40.0). In 1998, a new calendar of prophylactic immunizations was implemented based on the Ministry of Health Order No. 375 dated 18 December 1997, for the first time including vaccination of newborns against HB. Immunization of adolescents at 13 years of age was added to the schedule in 2001. Mass immunization of the Russian population was started as part of a nationwide program on 1 January 2006. 7 January 2010, a total of 45 million adults aged 18–59 were immunized, constituting almost 1/2 of the entire adult population of Russia [2].

HB immunization in children under 1 year old in Russia is performed in three doses: the first dose is injected within 24 hours of birth (in maternity hospitals), the other two doses are administered at 1 and 6 months.

In 2008, 10 years after the start of the HB vaccination campaign in Russia, the incidence of acute hepatitis B (AHB) was 4.0 per 100,000 people, further declining to 1.3 per 100,000 by 2014. However, the incidence of chronic hepatitis B (CHB) in Russia remains a source of concern: incidence of CHB in 2008 was 14.2 per 100,000 people, and in 2014, 11.3 per 100,000 [3]. This indicates a persistent, substantial reservoir of the infection. Nonetheless, data on the prevalence of HBV in the general population in the context of universal vaccination are scarce.

Given ongoing mass vaccination, HBV genetic variability is of high importance. Due to the absence of proofreading activity of HBV reverse transcriptase, the viral genome has a very high variability rate, the highest among all DNA viruses [4]. “Immune escape” mutations in the HBV genome, which allow the virus to evade vaccine-associated immune response, impart a selective advantage in the context of mass HB vaccination [5]. Therefore, surveillance of such mutations during long-term immunization campaigns is critical for assessing potential risks to immunization effectiveness and outcomes.

The aim of our study was to estimate the effect of vaccination on HBV-associated morbidity using a mathematical model based on epidemiological data, and to assess the prevalence of HBV immune escape variants after 10 years of vaccination.

## Materials and Methods

**Sera samples** were obtained from 6,217 volunteers in six regions of Russia in 2008, 10 years after initiation of mass immunization of newborns against HB. The volunteers comprised first time blood donors and patients of polyclinic and inpatient units of healthcare establishments without infectious diseases. Each participant completed a questionnaire with data about age, sex, socioeconomic conditions, presence of risk factors for HBV infection, and HBV vaccination status. Informed consent for scientific research was obtained in each case.

The study was conducted according to the principles expressed in the Declaration of Helsinki. Written informed consent was obtained from all participants. The study design was approved by the Ethics Committee of the Chumakov Institute of Poliomyelitis and Viral Encephalitides, Moscow, Russia (Approval #6 from 2010-04-01).

**Fig 1:**
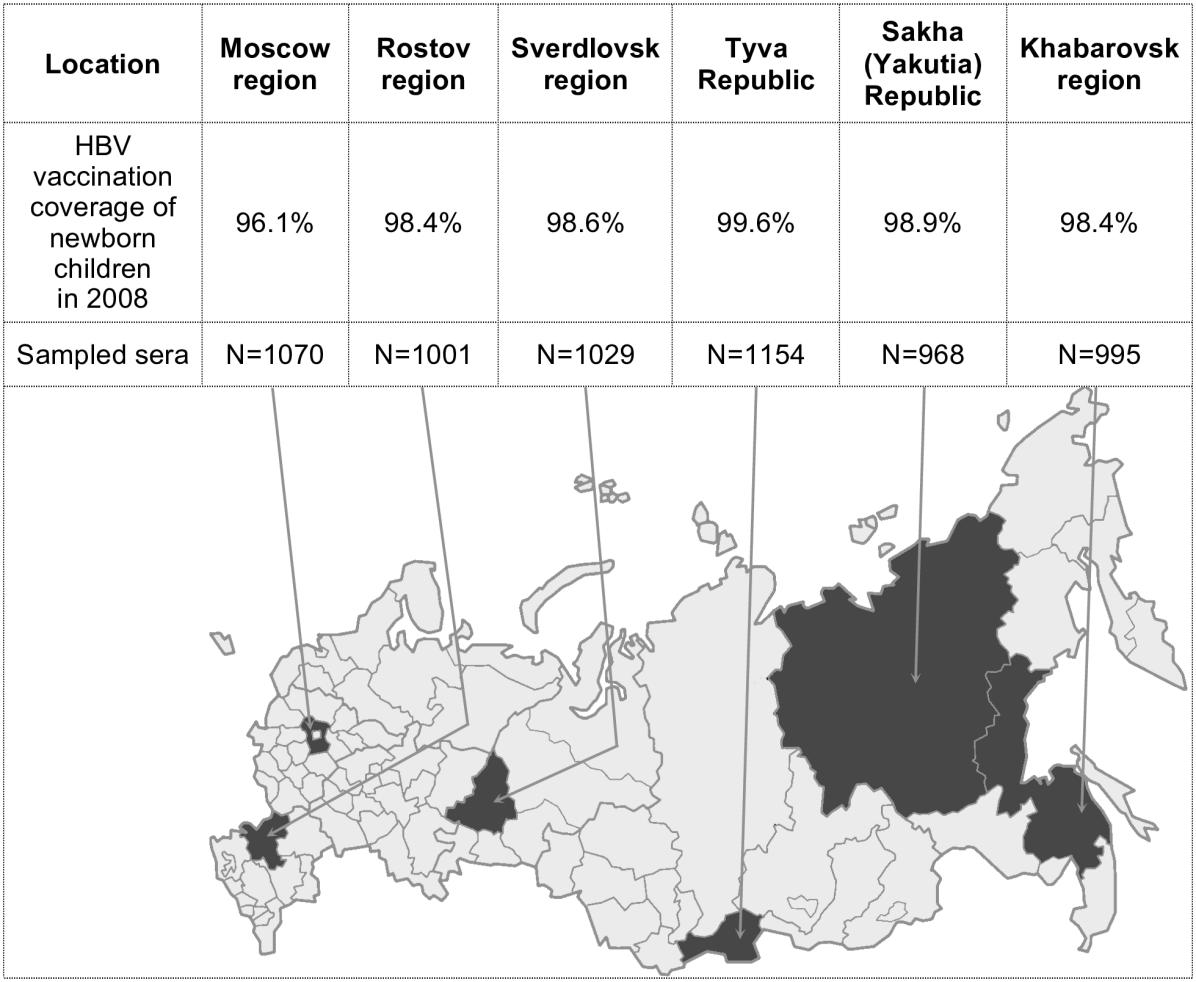
Sampling territories (shaded on the map), HBV vaccination coverage of newborn children in 2008, the number of sampled sera. Vaccination against hepatitis B includes three doses of vaccine. The first dose is administered to newborns in maternity wards in the first 24 hours after birth, the second dose in 1 month, and the third in 6 months.

The study included six geographically distant regions of Russia (Moscow, Rostov, Khabarovsk and Sverdlovsk regions, and Tyva and Sakha [Yakutia] Republics), which differ in HB incidence. Number of samples from each region is provided in Fig 1.

The study included all age groups, from children under 1 year to seniors over 60 years (<1 year, 1–4, 5–9, 10–14, 15–19, 20–29, 30–39, 40–49, 50–59, and ≥60 years). The mean population sample size in each age group was 104 individuals (54–151). The male/female ratio was 1:1.5.

A mathematical model developed at the Centers for Disease Control and Prevention (CDC) in the United States by Goldstein et al. in 2005 [6] was used to assess the impact of HBV vaccination on morbidity and rate of adverse outcomes.

The model is based on the following indicators: the prevalence of HBsAg and HBeAg in women of childbearing age (15–49 years); the prevalence of HBsAg and anti-HBc in two age groups (in children ≤5 years old and in adults ≥30 years old); the number of born children surviving the first year of life in the study year (2008); HBV vaccination coverage in children (three doses of the vaccine, with the first dose administered in the first 24 hours of life); and the efficacy of the vaccine used.

**Serological markers** of HBV (HBsAg, anti-HBc, HBeAg, anti-HBe) were determined by commercial ELISA kits (Diagnostic Systems, Russia). The sensitivity of HBsAg detection indicated by the kit manufacturer was 0.01 ng/ml.

**Detection, genotyping, and mutation analysis** of HBV DNA was carried out using PCR and subsequent direct sequencing of amplified S-region. HBV DNA testing was performed for all HBsAg and/or anti-HBc positive samples. DNA was extracted from 140 μl of serum samples using QIAamp Viral RNA Mini Kit (QIAGEN). HBV DNA detection was carried out in nested PCR with primers for overlapping S and P genes adapted from A. Basuni and W. Carman [7]. The sensitivity of HBV DNA detection in this in-house PCR assay was 100 copies/ml based on the results of serial dilution testing for PCR standards with known viral load.

Cycling conditions for PCR were as follows: 94°C for 2 min, 35 cycles at 94°C for 45 sec, 55°C for 45 sec, and 72°C for 90 sec; final elongation was performed at 72°C for 7 min. Size of the amplified product was 713 bp.

The 713-bp amplification product was excised from gel and purified from agarose using QIAquick Gel Extraction Kit (QIAGEN) following the manufacturer’s protocol. Sequencing was performed on a CEQ 8800 analyzer (Beckman Coulter, USA) using a Genome Lab Methods Development kit (Beckman Coulter, USA). The sequences were genotyped by comparison with reference sequences for A–H genotypes of “wild-type” HBV and checked for the presence of immune escape mutations in the HBsAg “a” determinant by comparison with the GenBank sequences carrying such mutations.

Statistical analysis of the results was performed usingcommonly used variational methods in Excel 2003 (Microsoft, USA) and GraphPad Prism 4 (GraphPad Software, USA). Statistical analysis included assessing the significance of differences of mean values between groups using Fisher's exact test and chi-squared distribution with Yates correction (significance threshold p<0.05).

## Results

### Prevalence of HB markers

**Fig 2:**
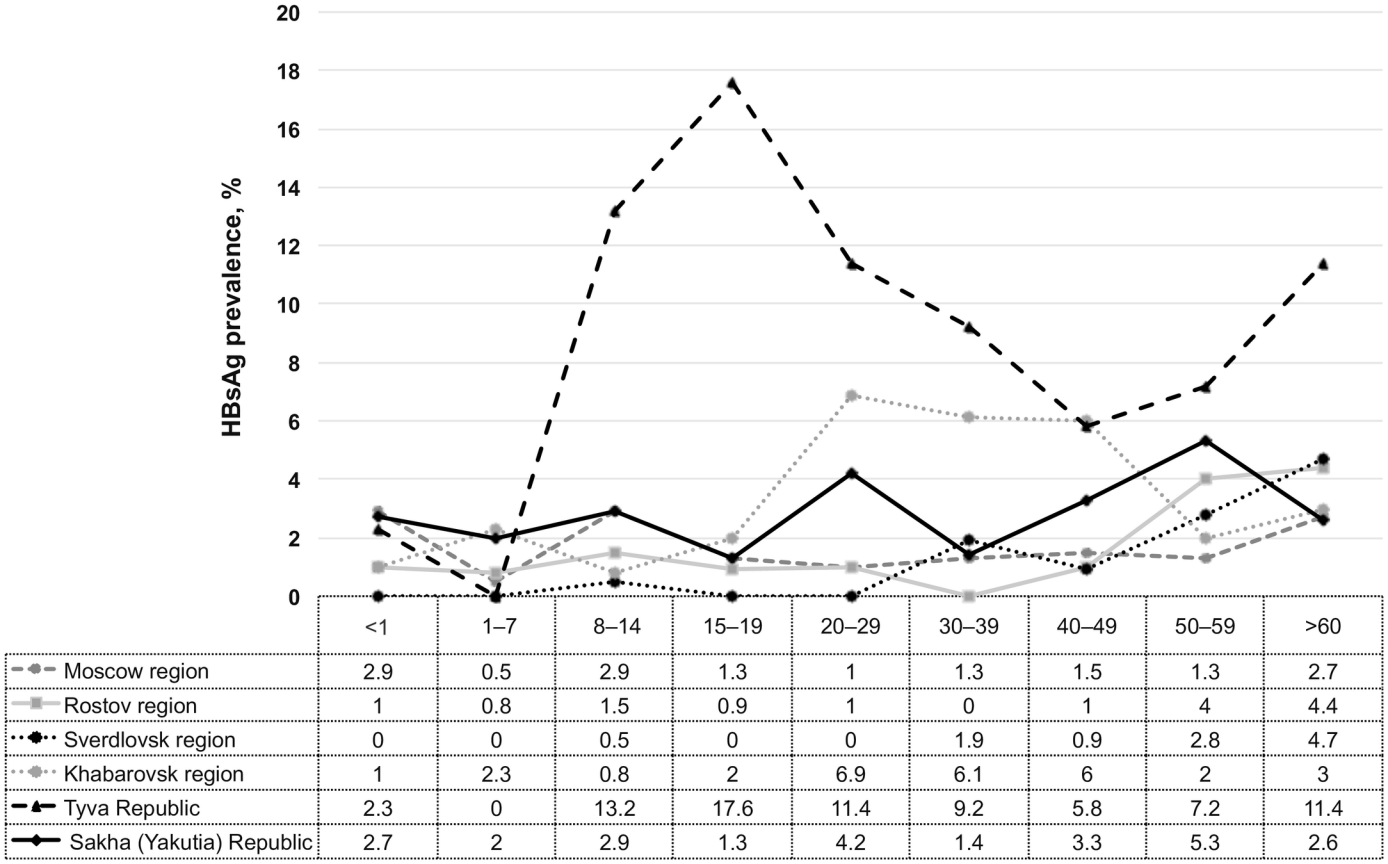
Prevalence of HBsAg in different age groups in six regions of Russia. In the age groups <1 and 1–7 years no significant differences in HBsAg prevalence between studied regions were observed. In age groups 8–14 and 15–19 HBsAg prevalence was significantly higher in Tyva Republic compared to other regions. In the age groups 20–29 and, 30–39 HBsAg rates were significantly higher in Tyva Republic and Khabarovsk region than those in other regions. In the groups > 60 years HBsAg prevalence in Tyva Republic was significantly higher than in Moscow region, Khabarovsk region and Sakha Republic (Yakutia).

HBsAg was detected in samples from almost all age groups in all studied regions of Russia. In Moscow region, mean prevalence of HBsAg in all age groups was 1.6%; in Rostov region, 1.8%; in Sverdlovsk region, 1.2%; in Khabarovsk region, 3.2%; in Tyva Republic, 8.2%; and in Sakha (Yakutia) Republic, 2.9%. Fig 2 shows the distribution of HBsAg in different age groups.

**Fig 3:**
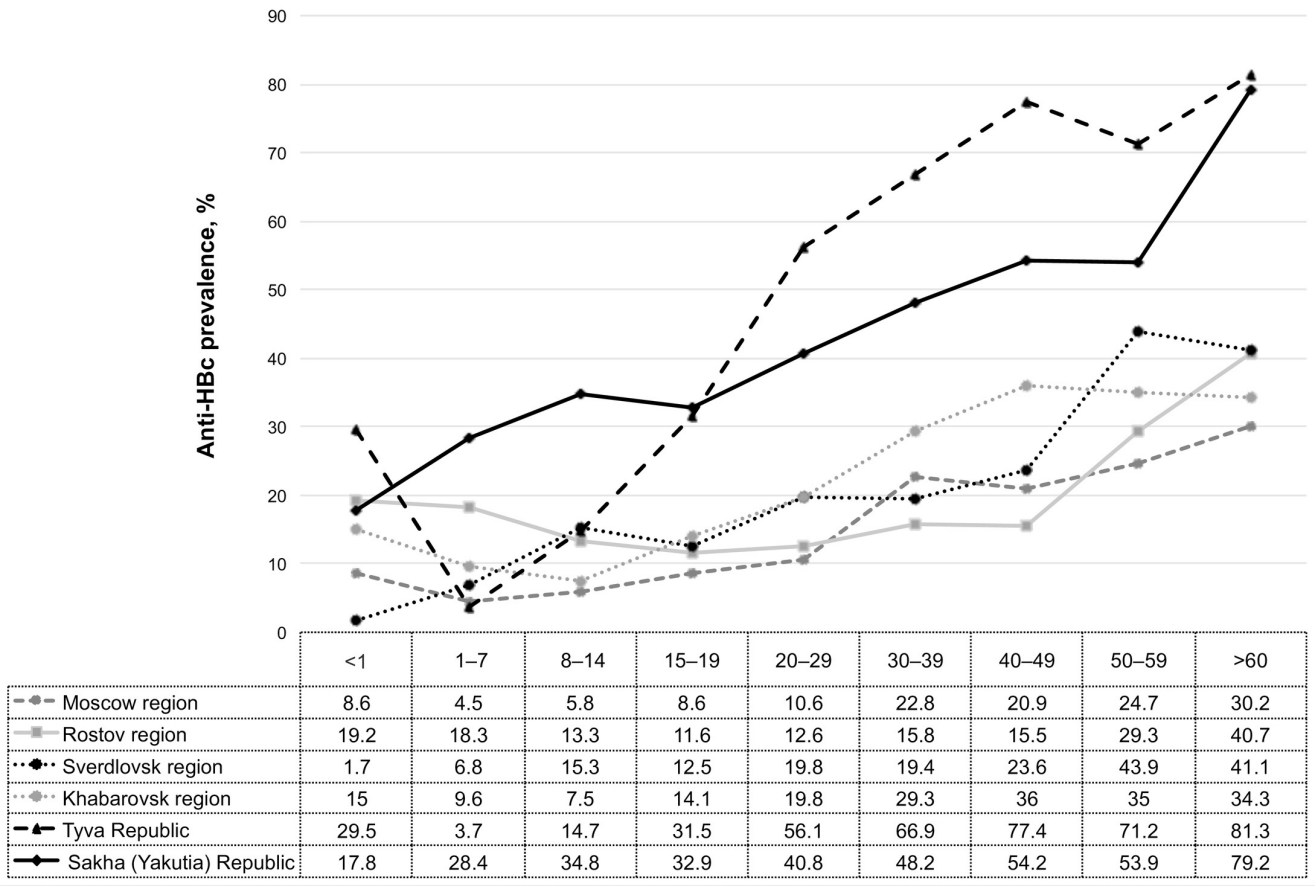
Prevalence of anti-HBc in different age groups in six regions of Russia. In the age groups <1 the rate of anti-HBc positivity was significantly higher in Tyva Republic compared to Moscow region, Sverdlovsk region, and Khabarovsk region. In the age groups 1–7 and 8–14 anti-HBc prevalence was significantly higher in Sakha Republic (Yakutia) compared to other regions. In the age groups 15–19, 20–29, 30–39, 40–49, 50–59, and > 60 years anti-HBc rates were significantly higher in Tyva Republic and Sakha Republic (Yakutia).

The prevalence of anti-HBc in Tyva Republic and Sakha (Yakutia) Republic was significantly higher (46.2% and 42.5%, respectively; p<0.05) than in the other studied regions: the equivalent value for Moscow region was 13.6%, Rostov region 18.9%, Sverdlovsk region 17.5%, and Khabarovsk region 21.0%. Fig 3 shows the distribution of anti-HBc in different age groups.

**Fig 4:**
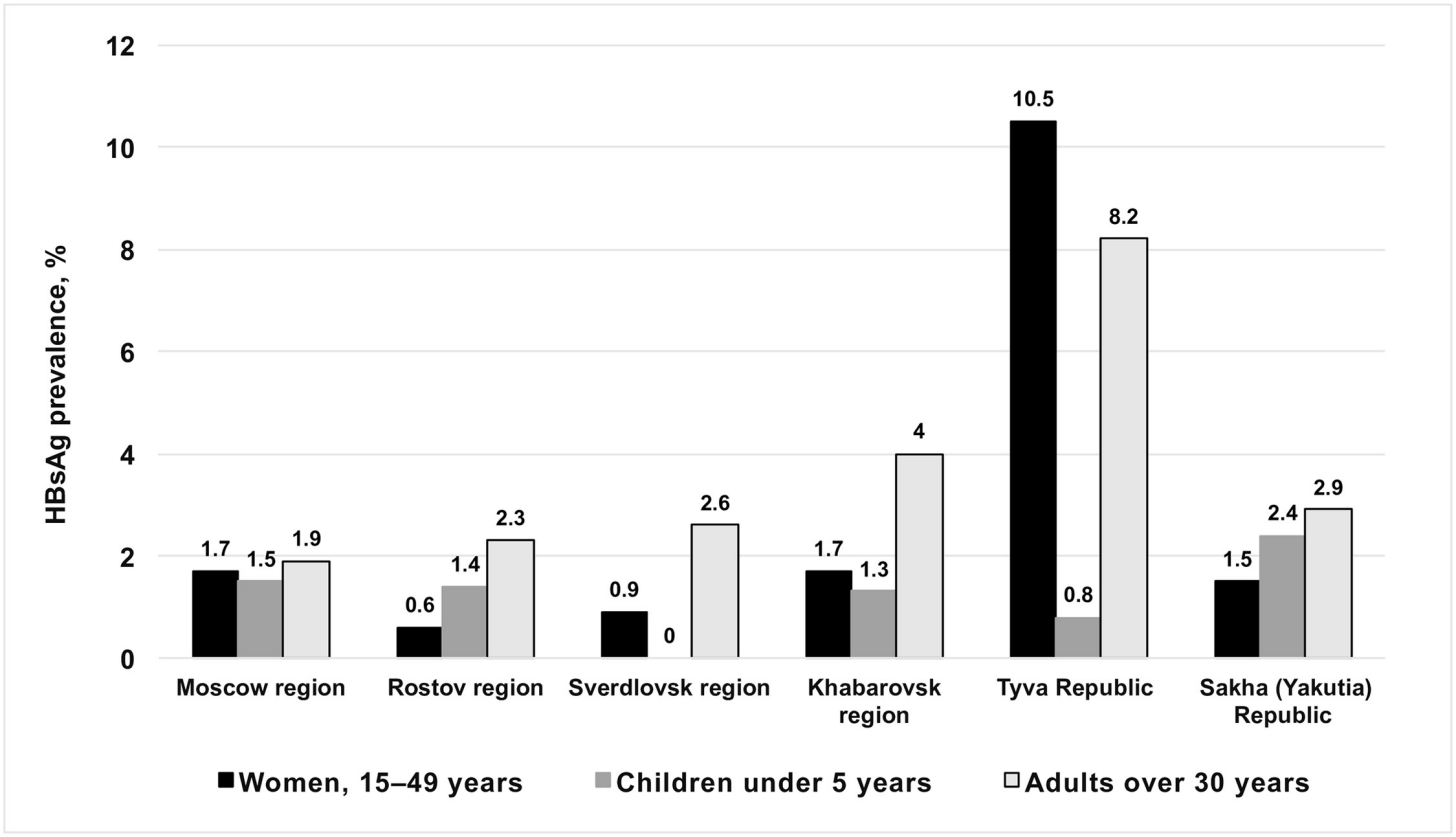
Prevalence of HBsAg in epidemiologically significant cohorts. These groups are considered to be epidemiologically significant as rates of HBV chronicity depend on the age of infection. In the perinatal infection chronicity of HBV infection occurs in 90% of cases, infection in early childhood—in 30% of cases, infection at the age of 5 years—6% [6]. Data on the prevalence of HBsAg in these groups needed to work with a mathematical model.

One of the parameters required in the mathematical model is the prevalence of HBsAg in epidemiologically significant subgroups: women of childbearing age (15–49 years), children under 5 years, and adults over 30 years. Such groups are used as rate of HBV chronicity depends on the age at which infection occurred [6]. For that reason, besides assessing prevalence of HB markers in the general population, we determined the prevalence of HBsAg in epidemiologically significant subgroups: women of childbearing age (15–49 years), children under 5 years, and adults over 30 years (Fig 4).

Prevalence of HBsAg in women of childbearing age in studied regions (Fig 4) was 0.6–1.7%, with the exception of Tyva Republic, where prevalence was significantly higher (10.5%; p<0.05). HBeAg in HBsAg-positive women of childbearing age was detected only in Tyva Republic (3.9%). The same was observed in the population aged ≥30 years: 8.1% in Tyva Republic compared to 1.9–4.0% in the other five regions (p<0.05).

Among children under 5 years (Fig 4), only Sverdlovsk region was free of HBsAg. In the other studied regions prevalence was 0.8–2.4%, without statistically significant differences between regions (p>0.05).

### Mathematical modeling of the impact of vaccination against HBV

**Fig 5:**
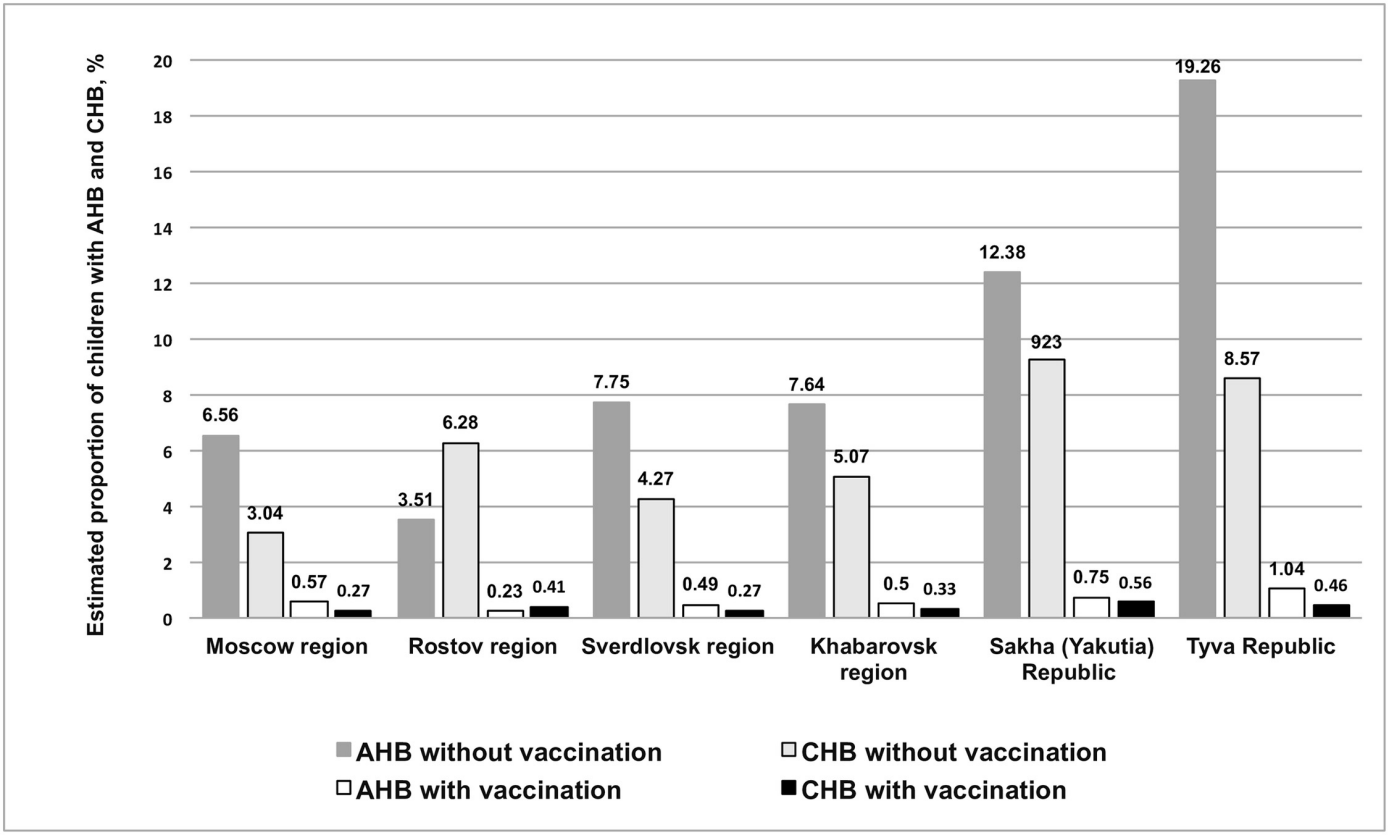
Estimated proportions of children with acute and chronic HB as a function of vaccination. Estimates are made using a mathematical model for a generation of children born in 2008.

We used a mathematical model with the data on the prevalence of HBV markers in epidemiologically significant subgroups obtained in our study, as well as published data on coverage with birth dose of HB vaccine in Russia, to estimate the number of AHB and CHB cases prevented by HB vaccination (Fig 5).

Based on the reported data on HBV vaccination coverage of newborn children in Russia (96.1–98.9%) [8], the number of expected AHB cases among the investigated generation (children born in 2008) can be reduced by vaccination 11.5–18.5 fold (11.5 in Moscow region; 15.4 in Rostov region, 15.8 in Sverdlovsk region, 15.3 in Khabarovsk region, 16.6 in Sakha (Yakutia) Republic, and 18.5 in Tyva Republic). The number of expected cases of CHB in the studied generation can be reduced 11.3–18.8 fold (11.3 in Moscow region, 15.3 in Rostov region, 15.7 in Sverdlovsk region, 15.3 in Khabarovsk region, 16.5 in Sakha (Yakutia) Republic, and 18.8 in Tyva Republic). Thus, we estimate that 91–95% of cases of AHB and CHB that would otherwise occur are, in fact, prevented by HBV vaccination (Fig 5).

Timely vaccination against HBV can reduce the estimated number of HB-associated deaths 11.1–18.5 fold (11.1 in Moscow region; 15.2 in Rostov region, 15.1 in Sverdlovsk region, 15.1 in Khabarovsk region, 15.7 in Sakha (Yakutia) Republic, and 18.5 in Tyva Republic) (Fig 5).

The largest effect on AHB and CHB cases and HB-associated deaths (18.5, 18.8, 18.5 fold; 95% overall reduction) is observed in Tyva Republic, which has the highest prevalence of HBsAg in the study population (8.2%) and the highest reported coverage of newborns with the first dose of HBV vaccine (99.6%) (Fig 5).

**Fig 6:**
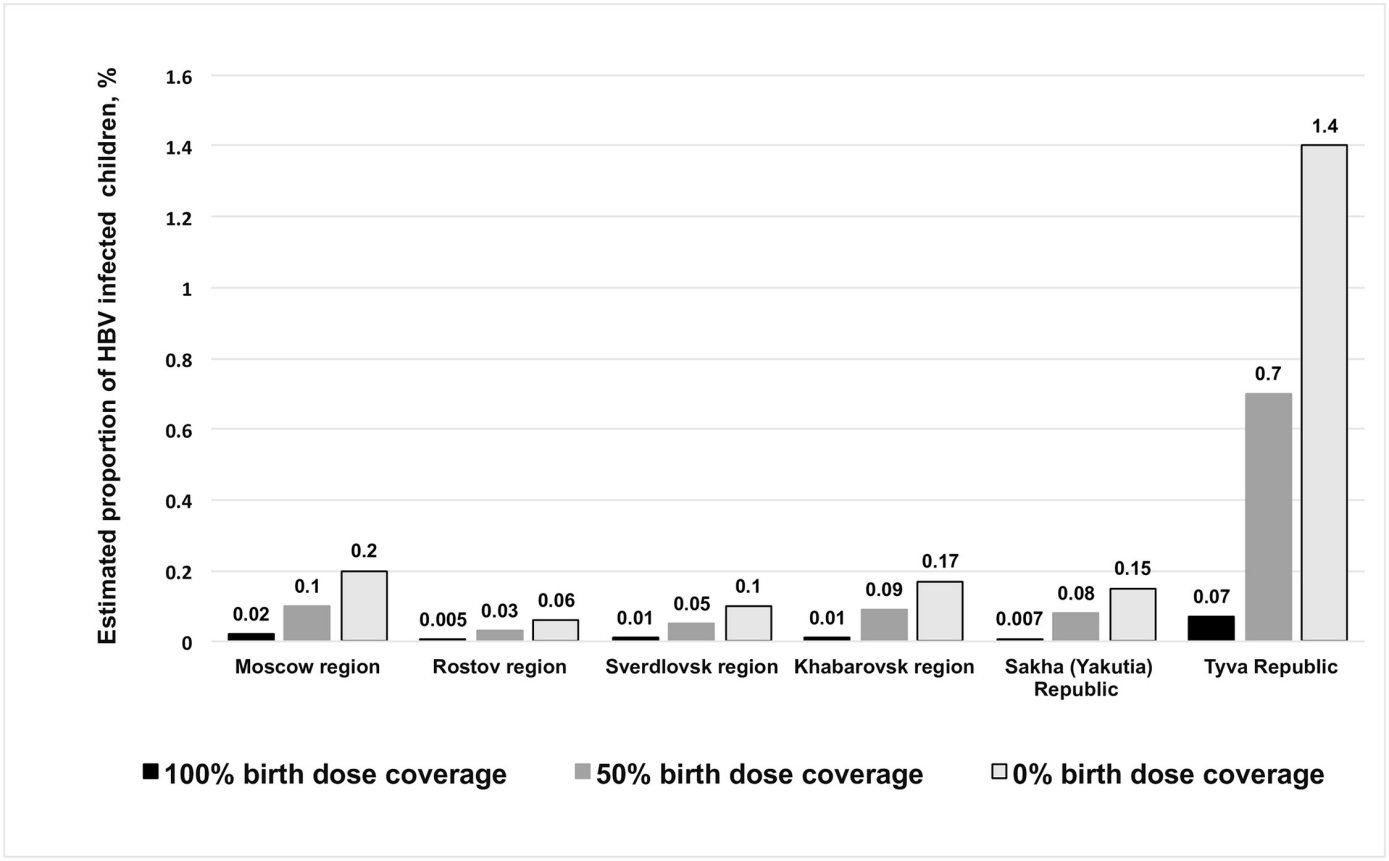
Estimated proportion of children infected with HBV depending on birth dose coverage scenario. Estimates are made using a mathematical model for a generation of children born in 2008.

Using the same model, we also estimated the influence of birth dose coverage (0%, 50%, and 100%) on the expected number of cases of HBV infection (Fig 6). Administration of a birth dose of HB vaccine to 50% of children is shown to reduce perinatal HBV infection cases 1.8–5.0 fold. Timely administration of the first dose to 100% of children reduces the estimated risk of perinatal HBV infection 10.0–21.4 fold. The greatest effect of birth dose coverage is observed in Tyva Republic, where the highest prevalence of HBsAg among women of childbearing age (10.5%) was detected.

### Prevalence of HBV immune escape mutants in HBsAg-positive individuals

HBV DNA was detected in 61 HBsAg-positive samples and in 2 HBsAg-negative anti-HBc-positive samples. Amplified fragments of viral DNA were sequenced, genotyped, and analyzed for the presence of mutations in the HBsAg coding region.

Distribution of HBV genotypes in HBV-infected individuals was as follows: genotype D was detected in 84% of studied samples (52/63), genotype A in 14% (9/63), and genotype C in 2% (1/63). In Moscow and Rostov regions, only genotype D was detected, while genotype C was found only in Khabarovsk region (Khabarovsk region borders China, where genotype C is highly prevalent). Unlike the other studied regions, in Sakha (Yakutia) Republic genotype A was more prevalent than genotype D (63% vs. 37%). Genotype D was represented by four subtypes: D1, D2, D3 and D4; genotype A had only subtype A2 and genotype C had only subtype C1.

For all the isolates of HBV in this study, serotype was determined based on the predicted amino acid sequence of HBsAg. Among the HBV samples from Moscow and Rostov regions, the *ay* serotype was detected in 100%; in Sverdlovsk region, 75%; in Khabarovsk region, 81.3%; in Tyva Republic, 95.2%; and in Yakutia, 37.5%. Serotype *ad* was detected in 62.5% of HBV samples from Sakha (Yakutia) Republic, 25% of samples from Sverdlovsk region, 18.7% of samples from Khabarovsk region, and 4.8% of samples from Tyva Republic.

Based on the analysis of HBV S-gene nucleotide sequences, we assessed the prevalence and number of amino acid substitutions in HBsAg. The prevalence of amino acid substitutions in HBsAg was 38% (24/63), but only 2 of these 24 mutations were within the “a” determinant of HBsAg (1 M133T mutation, 1 G145S mutation). The other 22 mutations were outside the “a” determinant (18 involving T118A/V, 4 involving Y206S/C) (Table 1). None of the mutations eluded HBsAg detection in the new-generation ELISA assays, since all samples carrying the mutations were HBsAg-positive in those assays.

**Table 1:** Amino acid substitutions within HBsAg in HBV strains isolated in Russia.

Number of HBV strains with aa substitutions (region of strain isolation)	Serotype/ genotype of HBV strains with aa substitutions	Detected aa substitutions / Variants described in literature	Phenotype of variants described in literature
**18 isolates** (Moscow region, Rostov region, Sverdlovsk region, Khabarovsk region, Tyva Republic)	1 isolate: *ayw2/D2*; 17 isolates: *ayw3/D2*	T118/V / T118/A	Change in antigenic properties of HBsAg / Product of selection in HBIG treatment [9]
**1 isolate** (Sakha [Yakutia] Republic)	1 isolate: *ayw2/D3*	M133/T / M133/H/L	Escape from HBsAg detection in ELISA assay [10]
**1 isolate** (Tyva Republic)	1 isolate: *ayw2/D3*	G145S / G145R/A/L	Escape from HBsAg detection in ELISA assays / Immune escape [10]
**4 isolates** (Moscow region, Rostov region, Sverdlovsk region, Khabarovsk region)	1 isolate: *ayw2/D2;* 1 isolate: *ayw2/D3;* 2 isolates: *ayw3/D2*	Y206S/C / Y206S	Change in antigenic properties of HBsAg / Product of selection in HBIG treatment [11]

## Discussion

Since Russian regions are diverse with respect to endemicity for HB, studies evaluating the impact of mass vaccination on public health must take these differences into account to produce results representative of the country as a whole. Therefore, based on retrospective analysis of data on CHB incidence, six geographically distant regions of Russia with differing CHB incidence were chosen: Moscow region, Rostov region, Sverdlovsk region, Khabarovsk region, Tyva Republic, and Sakha (Yakutia) Republic. Incidence of CHB was low (fewer than 6 cases per 100,000 population) in Moscow region and Rostov region; medium (6–15.9 per 100, 000) in Sverdlovsk region and Khabarovsk region, and high (more than 16 per 100, 000) in the republics of Tyva and Sakha (Yakutia) [3]. Differing incidence of CHB in these regions determines the difference in the number of potential sources of infection, which in turn affects the risk of contracting HBV.

Assessment of endemicity of different regions with respect to HB is performed based on HBsAg detection rates in the population. Regions with HBsAg detection rates over 8% are considered hyperendemic, those with 2–7% rates are moderately endemic, and those with rates ≤2% are low endemic [12]. Based on this classification Moscow region, Rostov region and Sverdlovsk region are of low HB endemicity; Sakha (Yakutia) Republic and Khabarovsk region are of intermediate endemicity, and Tyva Republic is hyperendemic. Despite high incidence of HBsAg in women of childbearing age in the latter region (10.5%), this marker was detected in only 0.8% of children under 5 years, indicating that the campaign of HB vaccination of newborns is successful.

Another region, which demonstrated a high effectiveness of HB vaccination is Sverdlovsk Region. Ten years after the start of the vaccination program, no HBsAg-positive sample was identified in children under 5 years in this region.

Although the vaccination program all regions of Russia held fairly evenly with the same (recombinant) vaccine, there were some differences that could have a positive impact on the vaccination effectiveness in Tyva Republic and Sverdlovsk region. Vaccination campaign in Tyva Republic was started in 1997, i.e. one year earlier than in all other regions of Russia. In both regions vaccination coverage > 90% of newborns was achieved rapidly. In Sverdlovsk region vaccination of adolescents 13–19 years old was started simultaneously with newborn vaccination [8].

After the initiation of mass HB vaccination in Russia, there was a more than 33-fold reduction in the number of AHB cases, from 43.8 per 100,000 in 1999 to 1.3 per 100,000 in 2014 [3]. Despite a significant decrease in the incidence of AHB and fewer new infections, there is still a large reservoir of infection in the general population due to CHB patients. In 2000–2011 the incidence of newly diagnosed CHB in Russia was 13 to 15 cases per 100 000 population, compared with 11.3 per 100,000 in 2014 [3]. This means that prevalence of HBV in the population, and therefore the risk of contracting the virus, remain high. Because the risk of chronic infection can be as high as 90% in perinatal infection, there is a need for early HBV prophylaxis. Mathematical modeling done in this study demonstrated that the current birth dose coverage level leads to a 10–21 fold increase in vaccination effectiveness compared to 50% and 0% birth dose coverage scenarios, depending on the epidemiological situation in a particular region. These results acquired from the model based on current Russian HB epidemiology data indicate the importance of >96% birth dose coverage for ensuring the continued effectiveness of the vaccination program.

Our calculations using the mathematical model developed by Goldstein et al. suggest that timely vaccination of newborns of the current generation has reduced the number of AHB and CHB cases by 91–95%. The best results are seen in Tyva Republic, which is hyperendemic for HB and has the highest reported coverage level of newborns with the first dose of HBV vaccine (99.6%) [8].

An analogous study performed using the same mathematical model for data obtained in Africa, the Americas, Middle East, South-East Asia, and Europe demonstrated that 90% vaccination coverage of newborns reduces the number of estimated cases of AHB and CHB by 84% [6]. The larger decline observed in our study is apparently due to higher vaccination coverage (96.1–99.6%) of newborns in the Russian Federation compared to the previously modeled countries, in which HB vaccination coverage does not exceed 90% [6,13]. Impact of HB vaccination, based on the model estimates, is stronger in regions with higher HBV prevalence and higher vaccination coverage.

Besides a reduction in morbidity, mass vaccination against HB may lead to an increase in the number of individuals harboring HBV variants with mutations in the “a” determinant of HBsAg associated with immune escape. Studies in Taiwan, where a vaccination campaign against HB has been ongoing since 1984, suggest a significant increase in the prevalence of HBV mutants [13]. Our results suggest that as of when the samples were collected (10 years after the start of the HB vaccination campaign), there was no significant increase in the prevalence of immune escape HBV mutants in the Russian Federation. One possible explanation of these findings is the fact that from the start of mass immunization in Russia, highly efficient recombinant (not plasma-derived) vaccines were used. It has been shown that mutant forms of HBV are detected significantly more often in individuals vaccinated with plasma-derived vaccines compared to those receiving recombinant vaccines [14]. Another possible explanation is that the rapid increase in the pace and broad coverage of mass vaccination against HB in combination with screening of donated blood has led to a significant decrease in the number of new infections, depriving the virus of opportunities to develop evolutionary adaptations.

## Conclusion

The necessity and the effectiveness of HB vaccination have been proven by the experience of many countries that have adopted universal vaccination, including Russia. Despite a large reduction in the incidence of AHB, the epidemic situation in regard of HB in Russia remains serious and requires continued efforts against HB with the first dose of the vaccine administered at birth. Furthermore, the low prevalence of HBV immune escape mutants 10 years after the start of mass vaccination offers a favorable prognosis for program effectiveness in the future.
